# Multichannel Recovery Potential with Activated Autologous Intraovarian Platelet-Rich Plasma and Its Derivatives

**DOI:** 10.3390/medicines10070040

**Published:** 2023-07-03

**Authors:** E. Scott Sills, Samuel H. Wood

**Affiliations:** 1 Regenerative Biology Group, FertiGen CAG, San Clemente, CA 92673, USA; 2 Department of Obstetrics & Gynecology, Palomar Medical Center, Escondido, CA 92029, USA; 3 Gen 5 Fertility Center, San Diego, CA 92121, USA

**Keywords:** ovary, aging, infertility, menopause, cell signaling, cytokines, PRP

## Abstract

Platelet-rich plasma (PRP) is an ‘orthobiologic’ with recognized roles in plastic surgery, musculoskeletal disorders, dentistry, dermatology, and more recently, ‘ovarian rejuvenation’. Intraovarian PRP involves a complex secretome discharged after platelet activation, comprising multiple cytokine mediators delivered surgically to older or inactive ovarian tissue. Loss of oocyte meiotic fidelity and impaired fertilization accompanying advanced maternal age are already managed by IVF, but only with eggs provided by younger donors. However, if the observed effect of rectifying embryo ploidy error can be proven beyond case reports and small series, activated PRP (or its condensed plasma cytokines) would deliver a welcome therapeutic disruption that is difficult to overstate. Because shortcomings in ovarian function are presently addressed mainly by pharmacological approaches (i.e., via recombinant gonadotropins, GnRH analogs, or luteal support), autologous PRP would represent an unusual departure from these interventions. Given the diversity of platelet cargo proteins, the target response of intraovarian PRP is probably not confined to oocytes or follicles. For example, PRP manipulates signal networks driving improved perfusion, HOX regulation, N-glycan post-translational modification, adjustment of voltage-gated ion channels, telomere stabilization, optimization of SIRT3, and ribosome and mitochondria recovery in older oocytes. While multichannel signals operating on various pathways are not unique to reproductive biology, in intraovarian PRP this feature has received little study and may help explain why its standardization has been difficult. Against this background, our report examines the research themes considered most likely to shape clinical practice.

## 1. Introduction

Tissue hypoxia broadly constrains organelle function. Any capillary enhancement via angiogenic factors from PRP would resonate with the effects observed experimentally in aged mice receiving hyperbaric oxygen therapy, where halted follicle apoptosis, improved oocyte maturation, and better fertilization were attained [1,2]. A comparable effect with intraovarian PRP was recently reported in a large animal veterinary model [3]. While free radicals accumulating from normal mitochondrial activity interfere with fertilization and embryo development, oocytes at early stages benefit by avoiding this stress using an electron transport chain (ETC) bypass to eliminate respiratory complex I [4]. While dampening downstream reactive oxygen species is one way PRP may help protect the mammalian ovary [5], verifying this in humans is difficult given the extreme scarcity of material for research. Indeed, early data generated from adult human ovarian tissue were only available from postmortem organ donation [6], where significant follicular development gain was observed when PRP was added to in vitro culture.

Mammalian stem cell homeobox (HOX loci) genes govern critical aspects of embryo somatic axial organization and can exhibit bivalent epigenetic potential, with both permissive and repressive features [7]. Immunohistochemical characterization of HOX cofactors in human ovarian tissue has revealed ‘temporal collinearity’ (i.e., time/place specific action) driven by growth differentiation factor-9 (GDF-9) and follicle-stimulating hormone (FSH) in granulosa cells [8,9]. Prominent HOXA7-positive staining is evident in follicles but is nearly absent in oocytes. Interestingly, granulosa cells of primordial follicles begin as predominantly HOXA7-negative, with uniformly HOXA7-positive nuclei in primary follicles. In this way, HOXA7 guides granulosa cell proliferation via epidermal growth factor (EGF) receptor regulation [10], and additional EGF signaling shared from autologous PRP may also interact at this level. Distribution of HOXA7 disperses as follicles mature from mainly nuclear to cytoplasmic [11], as HOXA7 expression is not seen on normal ovarian surface epithelium [12]. Another platelet cytokine, transforming growth factor-beta 1 (TGF-β1), independently promotes stemness traits [13].

## 2. Post-Translational Effects and Telomere Considerations

Adding to the understanding of how reproductive metabolism changes over time, several pathways cataloged in the Kyoto Encyclopedia of Genes & Genomes (KEGG) are preferentially upregulated in young murine follicles compared to older ovaries. Among these are N-glycan biosynthesis [14,15], a post-translational modification which determines immunogenicity, pharmacokinetics, and in vivo plasma protein clearance, as well as platelet function [16]. While Mercado et al. [17] were among the first to suggest that N-glycan surface features on platelets are influenced by 5-Hydroxytryptophan (5-HT), many 5-HT/serotonin-specific receptors are also known to be on oocytes and within ovarian tissue more generally [18]. Interactions between platelet cargo proteins and follicular surface markers following ovarian PRP invite interest because membrane dynamics determine cellular homeostasis via ion transport and modulation of key signaling pathways [19,20].

Numerous platelet growth factors have been considered in other tissues, with vascular endothelial growth factor (VEGF) being perhaps the best known, as it suppresses slowly activating delayed rectifier K+ current in cardiac cells [21]. Likewise, EGF is known to mimic OxyHb-induced voltage-gated potassium (K*v*) channel suppression in rabbit cerebral artery myocytes [22]. Another process, C-type inactivation, attenuates ion movement by rendering K*v* channels nonconductive [23,24]. Since ovarian K*v* channels in luteinized granulosa cell modulate cell production, proliferation, and apoptosis [25], discovering PRP-directed oocyte action at membrane interfaces would provide important clues to classifying how platelet cytokines may modify egg status.

**Figure 1:**
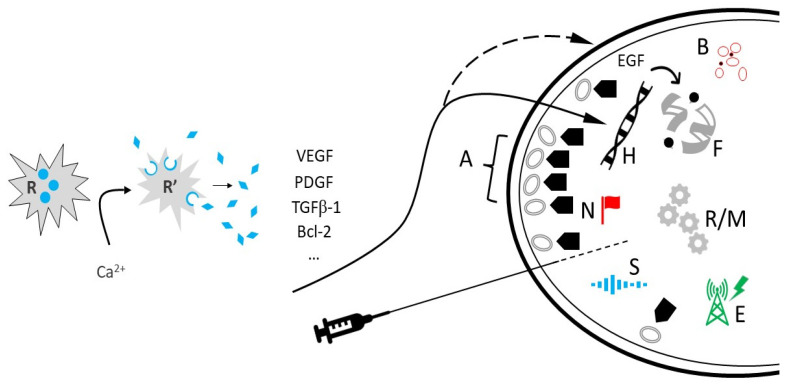
Fresh autologous platelets (R) are activated (R′) via calcium-based reagent, discharging numerous platelet-derived growth factors and cytokines available for intraovarian injection. EGF can exert a stabilizing effect on telomeres (F), and angiogenesis (B) promoted by VEGF permits general tissue support via improved perfusion. Regulators of HOX loci (H) may include upstream PRP elements, but stemness traits are partially under TGF-β1 control [38]. PRP components coordinate post-translational modifications, such as N-glycan biosynthesis (N) and optimization of SIRT3 (S), as well as improving mitochondrial/ribosomal metabolism (R/M). Ion channel regulation (E) may be an additional role for intraovarian PRP. While operating independent of latent subcapsular ovarian stem cells (A), PRP may deliver secondary actions (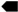) to commit undifferentiated progenitors to an oocyte lineage.

Telomere maintenance is essential for cell survival and basic homeostasis [26,27]. Not surprisingly, telomerase activity closely tracks ovarian reserve, as premature ovarian insufficiency is typified by sharply constrained telomerase activity [28]. Because EGF prompts telomerase activity most evident in smaller follicles with faster growth [29], its contribution to telomere function in ovarian tissue has received a careful inventory [30]. Besides supplying the template for telomeric DNA synthesis, telomerase RNA directly facilitates enzyme action via local reverse transcriptase to regulate the telomerase catalytic cycle [31]. While telomerase holoenzyme can build or replenish telomeric sequences by itself in vitro [32], additional protein factors are needed for proper recruitment and binding in vivo [33]. From this, the possibility that PRP might supply such accessory proteins has emerged as a compelling topic for research (see Figure 1).

For non-reproductive tissues, data on how platelets, cellular aging, and telomere status are functionally integrated are now accessible. For example, one large cross-sectional investigation recently described platelet traits, obesity indices, telomere length, and mitochondrial DNA copy number (mtDNAcn) [34]. In a sample of >5000 adults from rural Henan (China), increased central obesity indices were related to shorter telomere length or low mtDNAcn in association with abnormal platelet activity [34]. This clarifies the relation of overweight and platelet dysfunction [35], where risk for venous thromboembolism is increased by high mean platelet volume [36]. Yet, how platelets or their derivative growth factors (discharged upon activation) might affect telomerase activity is only beginning to be documented.

For example, Zhou et al. [37] identified the telomerase catalytic component hTERT as a transcription activator for vascular endothelial growth factor (VEGF), a known platelet releasate component [38]. While it is possible for this regulatory link to incorporate a feedback element (i.e., where VEGF influences telomerase function), this loop is incomplete. However, it has recently been shown that another product of activated platelets, insulin-like growth factor-1 (IGF-1), inhibits telomere shortening [39].

There are specific contexts where platelet function and telomere status are closely related. On this point, attention is drawn to the rare heritable condition, Zinsser–Cole–Engman Syndrome [40], where telomere abnormality is accompanied by low platelets and other features [40,41]. Clinical observations in reproductive medicine align with this pattern, as single-puncture ‘ovarian rejuvenation’ is less likely to recover an improved post-treatment serum anti-Mullerian hormone (e.g., AMH, as an ovarian reserve marker) when baseline platelet concentration is low [42]. The converse of this finding gained support from apparent blastocyst aneuploidy ‘correction’ following intraovarian PRP for a patient with an abnormally high platelet concentration [43]. Experts in New York [44] subsequently described a similar PRP procedure to yield improved embryo genetics in a study of 12 poor-prognosis IVF patients.

## 3. Is SIRT3 Replenished by PRP Components?

As a suite of regulatory enzymes organizing cell turnover, metabolism, and antioxidant protection, the sirtuins offer potential not just to reset ovarian function but, more dramatically, the promise of life extension [45]. The main deacetylase in mitochondria, sirtuin-3 (SIRT3) directs cellular energy and redox balance by buffering mitochondrial proteins via oxidized nicotinamide adenine dinucleotide (NAD+). While diminished SIRT3 action can lead to substantial aging pathologies, how it contributes to gonadal senescence was less clear until recently. Zhu et al. [46] measured substantially lower SIRT3 expression in ovarian tissue with advancing age. This discovery places age-related recession of SIRT3 in sharper focus for continued reproductive biology research.

Activators have been confirmed for some sirtuin isoforms, yet those specific to SIRT3 have only recently been isolated [47]. While ongoing work should verify if cytokines of platelet origin can directly modulate SIRT3, at least one corollary platelet process is established: IGF-1 promotes mitophagy and delays cellular aging via a nuclear respiratory factor-2 (NRF-2)/SIRT3-dependent pathway [39]. Since IGF-1 can reset mitochondrial membrane potential and cytochrome-C oxidase activity via NRF-2/SIRT3 [39], a comparable response in the ovary would not be surprising.

## 4. Subcellular Characteristics after PRP

Given the high translational activity of oocytes, there is little tolerance for perturbations in ribosome function or protein metabolism here [48]. Translational fidelity does tend to drop over time, setting the stage for maladaptive proteins unable to support normal development [15]. As oocyte nucleolar features undergo effacement during senescence, ribosome biogenesis is impacted with aging, but perhaps not in a way which would be expected [49]. For example, eggs from old mice have many more ribosomes than younger mice [15]. The absolute increase in ribosome count with advancing maternal age suggests a compensatory dial-up response to altered metabolic conditions. This has been similarly measured in mitochondria, where overall mtDNA level paradoxically rises with mitochondrial function decay [50]. In human blastocysts with mtDNA copy number exceeding an established threshold, this ominous sign forecasts poor reproductive outcome [51,52]. For example, mtDNA copy number is much higher for aneuploid embryos [53], illustrating a ‘less is more’ relation for human embryos and mtDNA copy number [54,55]. Further IVF experience with human embryo culture has indeed shown how mtDNA levels are usually far lower for cases with successful live birth [56].

In both instances, organelle adaptations which accompany cellular senescence tend to drive quantity upticks as a remedy to offset low quality. How might PRP intervene in this process? Wang et al. [57] recently showed both mammalian ribosomal protein S27a (a 40S ribosome subunit component) and ubiquilin-1 (a proxy marker for ribosome biosynthesis) are promoted by PRP, helping to explain experimental tissue regeneration effects observed after treatment. Another possibility is that PRP directly reinforces the ribosomal and mitochondrial complement by sharing platelet organelles (presumably of better quality) with recipient tissue. Operating separately from cytokines or other growth factors discharged after activation, any platelet ribosomes [58] or platelet mitochondria [59] contributed by intraovarian PRP have yet to be specifically tracked.

One major event during apoptosis is the escape of cytochrome-C (cyt-C), a highly conserved mitochondrial hemeprotein vital to electron transport and cellular respiration [60]. Transit of cyt-C into cytosol can occur through a membrane channel controlled by the first regulator of apoptosis to be identified in any organism, Bcl-2.61 Bcl-2 thus exerts an anti-apoptotic function by conserving membrane potential via blocking release of cyt-C [61,62]. While PRP can induce proliferation of Bcl-2 with therapeutic effects resulting from significant declines in cyt-C [61], this awaits full characterization in the adult human ovary.

## 5. PRP and Organ Damage Reversal

When mice are experimentally rendered infertile by cyclophosphamide, treatment with conditioned medium and PRP was able to boost expression of ‘mothers against decapentaplegic’ homologs 1 and 2 (SMAD1, SMAD2), growth differentiation factor-9 (GDF9), and bone morphogenetic protein-15 (BMP15) [63]. Although synergy may be inferred with PRP and conditioned medium, PRP carries its own cytokines, including platelet derived growth factor (PDGF), GDF9, and TGF-β [64,65]. For human granulosa cells, TGF-β1 upregulates gap junction alpha-1 protein/connexin 43 (Cx43) to amplify intercellular communication specifically involving activin receptor-like kinase (ALK) 5-mediated SMAD-related proteins [66]. Because this connexin protein may initiate differentiation, proliferation, or apoptosis [66], it is too soon to map precisely how PRP organizes a therapeutic ovarian response.

Response to another PRP protocol in murine liver tissue severely fibrosed by γ-radiation/Pb(NO_3_)_2_ showed amelioration of profound hepatotoxicity by intravenous PRP [67]. The effect was mediated by upregulated extracellular signal-regulated kinase cascade 1/2 (ERK1/2) and protein kinase B (Akt) signal pathways, with reduced intracellular oxidation [67]. Such observations join other reports unrelated to reproductive biology [5,61,67] where metabolic spoliation was erased following PRP dosing. The question of similar PRP effects in the adult human ovary seems answered, for now, only by small series and case reports [42,43,44]. In the meantime, knowledge of how specific platelet activation reagents differentially orchestrate platelet exosomes—which work like nanoscale transport shuttles for mRNAs, growth factors, and other cargo proteins—is enlarging [68,69].

## 6. Conclusions

Widefield RNA-seq analysis can portray a variegated picture of reproductive aging with uneven gene expression profiles [15]. Follicles of aged mice do occasionally appear like those of much younger animals, akin to clinical IVF where not all eggs from the aged ovary have uniform reproductive potential [15]. From this, skepticism concerning published ovarian PRP results rightly underscores how any beneficial outcome might simply be a chance event—particularly when patient age is relatively low [70,71,72]. Of note, the largest published dataset on ovarian PRP (*n* = 510) reported on poor-prognosis patients up to age 45 yrs, and achieved embryo transfers for 65.8% of cases [73].

The pleiotropic nature of PRP adds another investigative dilemma and explains why recommending intraovarian PRP is premature, outside of research settings, for fertility restoration [74,75]. As experience with intraovarian PRP grows, a signature for slowing the pace of reproductive aging is becoming less obscure. Notwithstanding proposals for how new (and better) eggs may be coaxed directly from latent ovarian stem cells by PRP [76], the current discussion presents multiple other routes where platelet cargo proteins could enable desirable results. Effective treatment alternatives would fill a ready niche in women’s health, as older patients with absent or low ovarian reserve generally bring the poorest prognosis. It is encouraging that the ovary is not the only complex organ where benefits have been achieved, as pancreatic function has also been successfully recovered with PRP [77]. While current infertility practice already includes technologies which received acceptance without evidence-based backup [78], this does not mean more should be welcome. Intraovarian PRP thus enters the field of fertility ‘add ons’ [79] as *tandem inter pares*, with much still to prove.
